# Effectiveness of the AS04‐adjuvanted HPV‐16/18 vaccine in reducing oropharyngeal HPV infections in young females—Results from a community‐randomized trial

**DOI:** 10.1002/ijc.32791

**Published:** 2019-12-14

**Authors:** Matti Lehtinen, Dan Apter, Tiina Eriksson, Katja Harjula, Mari Hokkanen, Tuomas Lehtinen, Kari Natunen, Silvia Damaso, Maaria Soila, Dan Bi, Frank Struyf

**Affiliations:** ^1^ Department of Infections and Cancer German Cancer Research Center (DKFZ) Heidelberg Germany; ^2^ Department of Laboratory Medicine Karolinska Institute Stockholm Sweden; ^3^ VL‐Medi Helsinki Finland; ^4^ Faculty of Social Sciences University of Tampere Tampere Finland; ^5^ GSK Wavre Belgium; ^6^ GSK Espoo Finland

**Keywords:** human papillomavirus, oral infection, oropharyngeal cancer, vaccine effectiveness

## Abstract

We studied effectiveness of the AS04‐adjuvanted HPV‐16/18 (AS04‐HPV‐16/18) vaccine against human papillomavirus (HPV) oropharyngeal infections associated with the increase of head/neck cancers in western countries. All 38,631 resident adolescents from 1994 to 1995 birth cohorts of 33 Finnish communities were invited in this community‐randomized trial (NCT00534638). During 2008–2009, 11,275 girls and 6,129 boys were enrolled in three arms of 11 communities each. In Arm A, 90% of vaccinated girls/boys, and in Arm B, 90% of vaccinated girls received AS04‐HPV‐16/18 vaccine. Other Arm A/B and all Arm C vaccinated participants received control vaccine. All Arm A participants and Arm B female participants were blinded to vaccine allocation. Oropharyngeal samples were analyzed from 4,871 18.5‐year‐old females who attended follow‐up visit 3–6 years postvaccination. HPV DNA prevalence was determined by SPF‐10 LiPA and Multiplex type‐specific PCR. Total vaccine effectiveness (VE) was defined as relative reduction of oropharyngeal HPV prevalence in pooled Arms A/B HPV‐vaccinated females *vs*. all Arm C females. VE against oropharyngeal HPV‐16/18, HPV‐31/45 and HPV‐31/33/45 infections were 82.4% (95% confidence intervals [CI]: 47.3–94.1), 75.3% (95%CI: 12.7–93.0) and 69.9% (95% CI: 29.6–87.1), respectively. In conclusion, the AS04‐HPV‐16/18 vaccine showed effectiveness against vaccine and nonvaccine HPV‐types oropharyngeal infections in adolescent females up to 6 years postvaccination.

## Abstract 2

**What's new?**

The incidence of oropharyngeal cancers due to human papillomavirus (HPV) infection is increasing in Western countries. However, the efficacy of HPV vaccines against oropharyngeal infection is not well documented. Based on a large community‐randomized trial in Finland, the authors report high effectiveness (82.4%) of *Cervarix* vaccine against oropharyngeal HPV‐16/18 infection and moderate effectiveness against non‐vaccine types HPV‐31/33/45 infection in female adolescents. The protective effects were observed up to 6 years after vaccination. These results provide further evidence that HPV vaccination holds the potential to reduce oral HPV infection, thereby offering protection against HPV‐related head and neck cancers.

**Abbreviations**
Al: aluminum
AS04‐HPV‐16/18: AS04‐adjuvanted HPV‐16/18 vaccine
CI: confidence interval
GCP: good clinical practice
HBV: hepatitis B virus
HPV: human papillomavirus
hrHPV: high‐risk human papillomaviruses
IARC: International Agency for Research on Cancer
MPL: monophosphoryl lipid A
*n* (%): number (percentage) of subjects reporting an event
*N*: number of subjects with available results
OPSCC: oropharyngeal squamous cell cancer
VE: vaccine effectiveness

## Introduction

Oncogenic, high‐risk human papillomaviruses (hrHPV) cause worldwide 9% of cancers in females and 1% in males.^1^ More than 15 years ago, a retrospective case‐series and a large nested case–control study showed that infection with HPV‐16 is associated with a high risk of oropharyngeal squamous cell cancer (OPSCC)^2^, ^3^ that develops 10 or more years after acquisition of the infection.^3^ Identification of HPV DNA in oropharyngeal and tonsillar tumor tissues have confirmed these observations.^4^, ^5^ In 2009, the International Agency for Research on Cancer (IARC) recognized the causal role of HPV‐16 in the etiology of cancers of the oropharynx and tonsil.^6^ The HPV association is most true for lymphoepithelial associated OPSCC‐subsites^5^ infected with HPV.

In western countries, the incidence of HPV‐positive OPSCC has increased very rapidly, virtually doubling in a decade since the 1970s.^7^, ^8^ Such trends have not been observed in HPV‐negative OPSCC. The former is clinically significantly more favorable^9^ and can be identified/monitored using HPV early antigen antibodies.^10^ Thus, HPV testing is important but prospects for screening of HPV‐positive OPSCCs may not be optimal due to the lack of defined manageable precancerous lesions. Moreover, in vaccinated populations with low background prevalence the predictive values for any screening test^11^ will be demanding.

In the adolescent and young adult populations, the prevalence of oral HPV infections varies widely from 0.4% to 10% mostly depending on the method used for viral DNA detection.^12^, ^13^, ^14^, ^15^ While HPV vaccination induces antibody levels at oral cavity that correlate with circulating antibody levels,^16^, ^17^ the efficacy of prophylactic HPV vaccines against oral infections has not been extensively studied. Up to 88% reduction of oral hrHPV infections in vaccine recipients as compared to nonvaccinated adolescents was recently suggested^18^ but randomized trial data are scarce.^12^ Based on a large community‐randomized trial^19^ we now report secondary objective results on the total vaccine effectiveness (VE) of the AS04‐adjuvanted HPV‐16/18 (AS04‐HPV‐16/18) vaccine against oropharyngeal HPV infections in adolescent females in Finland.

## Materials and Methods

### Community randomization

All the 33 randomized Finnish communities had more than 35,000 inhabitants.^19^ HPV‐16/18 seroprevalence rates by community were estimated based on sera from the population‐based Finnish Maternity Cohort.^19^ Each study arm comprised 4 high (>24%), 3 intermediate (24–20.5%) and 4 low (<20.5%) HPV‐16/18 seroprevalence communities. HPV seroprevalence is a measure of the cumulative HPV incidence, and as such gives a valid estimate of the background HPV exposure. This reduced the coefficient of variation across arms, and increased statistical power.^19^

### Ethics

The primary HPV‐040 trial (NCT00534638, clinicaltrialgov.com) and its amendment for the collection of the oral gargle samples were approved by the ethical committee of the Pirkanmaa hospital district in 2007 and 2011. The trial was conducted in accordance with good clinical practice (GCP) and all applicable regulatory requirements including the Declaration of Helsinki. Participants aged <15 years provided written informed assent; written informed consent was obtained from the participants’ parents or legal representatives. Written informed consent was obtained from study participants aged 15 years in line with local regulations.

### Study procedures

In the 2008–2009 school year, invitation letters were sent to the parents or legal guardians of all the 80,272 Finnish or Swedish speaking 1992–1995 born adolescents residents in the 33 communities identified by the Finnish Population Information System maintained by the Population Register Centre. The letters included trial information, parental and adolescents’ consent forms and a prepaid return envelope.

The vaccination (first three study visits) took place at the healthcare facilities of 250 municipal junior high schools of the study site communities. In Arm A, 90% of vaccinated participants received *Cervarix* (AS04‐HPV‐16/18; GSK) vaccine, and 10% received *Engerix B* vaccine (hepatitis B virus [HBV] vaccine; GSK).^19^ AS04 is an Adjuvant System containing monophosphoryl lipid A (50 μg MPL; produced by GSK) adsorbed on Aluminum salt (500 μg Al^3+^). In Arm B, 90% of vaccinated females received the AS04‐HPV‐16/18 vaccine, and 10% of vaccinated females and all vaccinated males received the HBV vaccine. The 90 and 10% proportions were receiver‐blinded proportions to assess the herd effect. The blinding was maintained for girls and boys in Arm A and for girls in Arm B. In Arm C communities, all vaccinated participants received the HBV vaccine. Virtually all (99%) vaccinated participants received all three vaccine doses.

Both vaccinated and nonvaccinated females of the 1994–1995 birth cohorts from the study site communities were invited to attend follow‐up visit at the age of 18.5 years during 2012–2014. Cervical and oropharyngeal samples for HPV DNA testing were obtained on each visit. Setting the age for attending the follow‐up visit at 18.5 years was per protocol to allow a minimum of 3 years between vaccination and cervical sampling.^19^

During the follow‐up visit, the attendees filled‐in a questionnaire on living conditions, life‐habits and sexual behavior. At age 18.5 years, a cross‐vaccination with either the HBV vaccine or the AS04‐HPV‐16/18 vaccine was offered to the attendees. Female attendees (139), who had moved between Arm C and Arm A or B communities during the follow‐up by age 18.5 years were removed from the final analyses.

### Laboratory analyses

All samples were analyzed by PCR for HPV DNA. HPV typing was performed by a broad‐spectrum PCR SPF10‐LiPA25 (Labo Biomedical Products, Rijswik, the Netherlands) using HPV‐specific hybridization probes enabling detection of 14 oncogenic HPV types (HPV‐16, 18, 31, 33, 35, 39, 45, 51, 52, 56, 58, 59, 66 and 68) and 11 nononcogenic HPV types (HPV‐6, 11, 34, 40, 42, 43, 44, 53, 54, 70 and 74). To ensure maximum sensitivity in the detection of HPV‐16, 18, 31, 33, 35, 39, 45, 51, 52, 56, 58, 59, 66, 68, 6 and 11, samples initially considered to be SPF‐10/DEIA positive for HPV were re‐evaluated by the multiplex type‐specific PCR.^20^

### Statistical analyses

The number of subjects invited to participate in the study and the number of subjects enrolled was tabulated by gender, for birth cohorts 1994, 1995 and overall.

The analysis of VE was done on female study participants and was based on the total enrolled cohort which included all study participants from all communities, including subjects who only completed the behavioral questionnaire at 18.5 years of age. For the analysis of a specific endpoint, only subjects with measured endpoints were considered.

The statistical analysis of VE of the AS04‐HPV‐16/18 vaccine against oropharyngeal infection was done by comparing the prevalence in females vaccinated with the AS04‐HPV‐16/18 vaccine from pooled Arms A and B over the prevalence in all females from Arm C for birth cohorts 1994–1995, for the following HPV types: HPV‐16, HPV‐18, HPV‐16/18, HPV‐6/11, HPV‐31/45 and HPV‐31/33/45.

The VE was computed as 1 minus the odd ratio of prevalence rates between the investigated arms and the control arm, estimated using the Mantel–Haenszel test adjusted for clustering and stratified by the historical seroprevalence used in the randomization (historical seroprevalence under 20.5%, between 20.5–24% and over 24%). The 95% confidence interval (CI) on VE was computed using the general inverse variance approach.

As a post hoc analysis, the VE of the AS04‐HPV‐16/18 vaccine against oropharyngeal infection for birth cohorts 1994–1995 was computed for the following HPV types: HPV‐31, HPV‐33, HPV‐35, HPV‐45 and HPV‐52. For this post hoc analysis, only point estimated of the VE was provided due to the low number of events. It was computed as 1 minus the prevalence (Supporting Information Table S1) in females vaccinated with the AS04‐HPV‐16/18vaccine from pooled arms A and B over the prevalence in all females from Arm C.

## Results

### Recruitment and follow‐up visit

Recruitment to the trial was population‐based and essentially balanced across all three study arms (Table 1). Also, attendance to the follow‐up visit at the age of 18.5 years by vaccinated female trial participants and nonvaccinated female residents of the different trial arm communities was similar.

**Table 1:** Number (%) of invited, enrolled and followed females by vaccination arm and birth cohort in 2008–2009* and 2012–2014 (follow‐up visit)

	Birth cohort		
Arm	1994	1995	Total
A			
Invited	3,063	2,846	5,909
Enrolled	1,964 (64.1)	1,660 (58.3)	3,624 (61.3)
Followed	831	775	1,606
B			
Invited	3,572	3,408	6,980
Enrolled	2,059 (57.6)	1,913 (56.1)	3,972 (56.9)
Followed	813	773	1,586
C			
Invited	3,040	3,027	6,067
Enrolled	1,890 (62.2)	1,789 (59.1)	3,679 (60.6)
Followed	846	833	1,679
All			
Invited	9,675	9,281	18,956
Enrolled	5,913 (61.1)	5,362 (57.8)	11,275 (59.5)
Followed	2,490	2,381	4,871

Arm A: 90% of vaccinated females and males received the AS04‐adjuvanted HPV‐16/18 (AS04‐HPV‐16/18) vaccine, 10% received the hepatitis B virus (HBV) vaccine. Arm B: 90% of vaccinated females received the AS04‐HPV‐16/18 vaccine, 10% of vaccinated females and 100% vaccinated males received the HBV vaccine. Arm C: 100% of vaccinated females and males received the HBV vaccine. The total enrolled cohort included all study participants from all communities, including subjects who only completed the behavioral questionnaire at 18.5 years of age. The followed females included all subjects with human papillomavirus (HPV) DNA Polymerase Chain Reaction result available for the oral gargle sample taken at the follow‐up visit and used in the computation of the total vaccine effectiveness. *Q1/2010, for the 1995 birth cohort.

### Vaccine effectiveness against oropharyngeal HPV infections

We found high to moderate VE against oropharyngeal hrHPV infections. VE against vaccine HPV types 16/18 was high (82.4%; 95% CI 47.3, 94.1), and indistinguishable between the two types (Table 2). VE against cross‐protected HPV types 31/45 and 31/33/45 was 75.3% (95% CI: 12.7, 93.0) and 69.9% (95% CI 29.6, 87.1), respectively.

**Table 2:** Total vaccine effectiveness against oropharyngeal infection with HPV in AS04‐HPV‐16/18 vaccinated females (pooled Arms A/B) *vs*. non‐AS04‐HPV‐16/18 vaccinated females (Arm C) for birth cohorts 1994–1995 vaccinated in 2007–2009 and attending follow‐up visits with oral gargle sampling in 2012–2014

	Arms A/B (*N* = 3,192)	Arm C (*N* = 1,679)	
HPV type	Positives *n* (%)	Positives *n* (%)	Arms A/B *vs*. Arm C VE % (95% CI)
HPV‐6/11	41 (1.3)	29 (1.7)	25.8 (−21.7, 54.8)
HPV‐16	6 (0.2)	19 (1.1)	81.3 (25.8, 95.3)
HPV‐18	4 (0.1)	10 (0.6)	78.9 (32.3, 93.4)
HPV‐16/18	9 (0.3)	27 (1.6)	82.4 (47.3, 94.1)
HPV‐31	2 (0.1)	8 (0.5)	86.8^1^
HPV‐33	6 (0.2)	9 (0.5)	64.9^1^
HPV‐35	3 (0.1)	3 (0.2)	47.4^1^
HPV‐45	1 (0.0)	1 (0.1)	47.4^1^
HPV‐52	14 (0.4)	12 (0.7)	38.6^1^
HPV‐31/45	3 (0.1)	9 (0.5)	75.3 (12.7, 93.0)
HPV‐31/33/45	9 (0.3)	16 (1.0)	69.9 (29.6, 87.1)

Arm A: 90% of vaccinated females and males received the AS04‐adjuvanted HPV‐16/18 (AS04‐HPV‐16/18) vaccine, 10% received the hepatitis B‐virus (HBV) vaccine. Arm B: 90% of vaccinated females received the AS04‐HPV‐16/18 vaccine, 10% of vaccinated females and 100% vaccinated males received the HBV vaccine. Arm C: 100% of vaccinated females and males received the HBV vaccine.

1 Only point estimated of VE was provided for this post hoc analysis due to the low number of events.

Abbreviations: CI, confidence interval; HPV, human papillomavirus; *N*, number of subjects with available results; *n* (%), number (percentage) of subjects reporting an event; VE, total vaccine effectiveness.

No VE was found against other hrHPV types (Table 2). VE estimate against the most notable low‐risk HPV types 6/11 was low (25.8; 95% CI −21.7, 54.8).

## Discussion

We found for the AS04‐HPV‐16/18 vaccine high VE against oropharyngeal infections with the vaccine HPV types 16 and 18 and a moderate VE against a prespecified combination of hrHPV types 31/33/45 not included in the vaccine. These results are in line with the reported, respectively high and moderate VEs of the AS04‐adjuvanted HPV‐16/18 vaccine against cervical HPV‐16/18 and HPV‐31/33/45 infections.^20^, ^21^, ^22^, ^23^

We hereby disclose results of our trial's secondary objective that was to evaluate VE of the AS04‐HPV‐16/18 vaccine against oropharyngeal infections in females of the 1994 and 1995 birth cohorts at the age of 18.5 years. Other objectives will be described in a separate publication and are available on the Clinical Trial Register (www.clintrialsgov.com; NCT00534638). The analyses related to the present objective are descriptive only.

For the targeted 1994–1995 birth cohorts, early vaccination age of 12–14 years at the baseline of our population‐based community‐randomized trial mimics national vaccination programs as does the female HPV vaccination coverage of approximately 50% in the HPV‐vaccination arms. The early age at vaccination was optimal for the vaccine‐induced immune response and to ensure the lowest possible proportion of baseline positives. High attendance rates of both originally vaccinated female participants and unvaccinated female residents at the age of 18.5 years retained the population‐based nature of our trial. This yielded robust VE estimates from the comparison of HPV‐16/18 vaccinated females in combined Arms A/B *vs*. non‐HPV‐16/18 vaccinated females in Arm C.

There was a long lag of 4–6 years in the collection of oral gargle samples postvaccination, while the oropharyngeal baseline HPV infection status in the study participants prior to vaccination was unknown. Identification of hrHPV types by highly sensitive PCR in the oral gargle is, however, the most comprehensive means for the identification of oropharyngeal HPV infections.^24^ Whether the identification of hrHPV type(s) in these cross‐sectional samples taken at the age of 18.5 years represents persistent infection can therefore not be judged, and must be considered as a limitation of the study. On the other hand, while 6 months and 12 months persistent positivity for the same HPV type has been used to define persistent cervical or other anogenital infections, very little is known about the duration of oropharyngeal infections. In the future, vaccine efficacy against oropharyngeal persistent hrHPV infection will be documented in an ongoing 10‐year follow‐up study with serial oral gargle sampling.

In conclusion, high VE against oropharyngeal infections with vaccine HPV types 16/18 and moderate VE against infections with nonvaccine HPV types 31/33/45 was observed in young adult females up to 6 years postvaccination with the AS04‐HPV‐16/18 vaccine. Our study provides further evidence that HPV vaccination could probably reduce oral HPV infections which may translate into protection against HPV‐related head and neck cancers.

## Supporting information


**Table S1** Prevalence of oropharyngeal infections, by individual oncogenic HPV type, Arm and Vaccine group, for birth cohorts 1994–1995 (Female study participants, Total enrolled cohort).

## Data Availability

Anonymized individual participant data and study documents can be requested for further research from www.clinicalstudydatarequest.com.
