# Supplementary material for: Effectiveness of the AS04‐adjuvanted HPV‐16/18 vaccine in reducing oropharyngeal HPV infections in young females—Results from a community‐randomized trial
Source: Int J Cancer. 2019 Dec 14;147(1):170–4. doi: 10.1002/ijc.32791 (PMC7318585; doi:10.1002/ijc.32791)
Supplement: Supplementary file 1 — Table S1 Prevalence of oropharyngeal infections, by individual oncogenic HPV type, Arm and Vaccine group, for birth cohorts 1994–1995 (Female study participants, Total enrolled cohort). [file IJC-147-170-s001.pdf]

Effectiveness of the AS04-adjuvanted HPV-16/18 vaccine in reducing oropharyngeal HPV infections in young females – results from a community-randomized trial

Matti Lehtinen, Dan Apter, Tiina Eriksson, Katja Harjula, Mari Hokkanen, Tuomas Lehtinen, Kari Natunen, Silvia Damaso, Maaria Soila, Dan Bi, Frank Struyf

<u>Table of content:</u>

- Supplementary table 1. Prevalence of oropharyngeal infections, by individual oncogenic HPV type, Arm and Vaccine group, for birth cohorts 1994-1995 (Female study participants, Total enrolled cohort).

Supplementary table 1. Prevalence of oropharyngeal infections, by individual oncogenic HPV type, Arm and Vaccine group, for birth cohorts 1994-1995 (Female study participants, Total enrolled cohort).

		Arm A				Arm B				Arm C			
**HPV Type**	**Group**	**N**	**n+**	**n**	**%**	**N**	**n+**	**n**	**%**	**N**	**n+**	**n**	**%**
6	AS04-HPV-16/18	1606	10	21	1.3	1586	9	15	0.9	-	-	-	-
	HBV	203	0	0	0.0	191	1	2	1.0	1446	10	19	1.3
	Not vaccinated	290	2	2	0.7	344	2	4	1.2	233	6	8	3.4
11	AS04-HPV-16/18	1606	3	3	0.2	1586	2	2	0.1	-	-	-	-
	HBV	203	1	1	0.5	191	1	1	0.5	1446	3	3	0.2
	Not vaccinated	290	1	1	0.3	344	0	0	0.0	233	2	2	0.9
16	AS04-HPV-16/18	1606	2	2	0.1	1586	3	4	0.3	-	-	-	-
	HBV	203	1	1	0.5	191	1	1	0.5	1446	8	13	0.9
	Not vaccinated	290	2	2	0.7	344	3	3	0.9	233	3	6	2.6
18	AS04-HPV-16/18	1606	2	3	0.2	1586	1	1	0.1	-	-	-	-
	HBV	203	2	2	1.0	191	1	1	0.5	1446	4	7	0.5
	Not vaccinated	290	2	2	0.7	344	2	2	0.6	233	3	3	1.3
31	AS04-HPV-16/18	1606	1	1	0.1	1586	1	1	0.1	-	-	-	-
	HBV	203	0	0	0.0	191	0	0	0.0	1446	4	6	0.4
	Not vaccinated	290	0	0	0.0	344	0	0	0.0	233	2	2	0.9
33	AS04-HPV-16/18	1606	2	2	0.1	1586	3	4	0.3	-	-	-	-
	HBV	203	0	0	0.0	191	1	1	0.5	1446	6	9	0.6
	Not vaccinated	290	2	3	1.0	344	2	3	0.9	233	0	0	0.0
34	AS04-HPV-16/18	1606	0	0	0.0	1586	1	1	0.1	-	-	-	-
	HBV	203	0	0	0.0	191	1	1	0.5	1446	0	0	0.0
	Not vaccinated	290	0	0	0.0	344	0	0	0.0	233	0	0	0.0
35	AS04-HPV-16/18	1606	1	2	0.1	1586	1	1	0.1	-	-	-	-
	HBV	203	0	0	0.0	191	0	0	0.0	1446	2	3	0.2
	Not vaccinated	290	0	0	0.0	344	0	0	0.0	233	0	0	0.0
39	AS04-HPV-16/18	1606	6	9	0.6	1586	4	6	0.4	-	-	-	-
	HBV	203	1	1	0.5	191	1	1	0.5	1446	5	6	0.4
	Not vaccinated	290	1	1	0.3	344	3	3	0.9	233	1	1	0.4
40	AS04-HPV-16/18	1606	1	1	0.1	1586	0	0	0.0	-	-	-	-
	HBV	203	0	0	0.0	191	0	0	0.0	1446	1	1	0.1
	Not vaccinated	290	0	0	0.0	344	0	0	0.0	233	1	1	0.4
42	AS04-HPV-16/18	1606	0	0	0.0	1586	0	0	0.0	-	-	-	-
	HBV	203	0	0	0.0	191	0	0	0.0	1446	0	0	0.0
	Not vaccinated	290	0	0	0.0	344	0	0	0.0	233	0	0	0.0
43	AS04-HPV-16/18	1606	2	3	0.2	1586	1	1	0.1	-	-	-	-
	HBV	203	0	0	0.0	191	0	0	0.0	1446	0	0	0.0
	Not vaccinated	290	1	1	0.3	344	0	0	0.0	233	1	1	0.4
44	AS04-HPV-16/18	1606	1	1	0.1	1586	3	3	0.2	-	-	-	-
	HBV	203	0	0	0.0	191	0	0	0.0	1446	0	0	0.0
	Not vaccinated	290	0	0	0.0	344	0	0	0.0	233	1	1	0.4
45	AS04-HPV-16/18	1606	0	0	0.0	1586	1	1	0.1	-	-	-	-
	HBV	203	0	0	0.0	191	0	0	0.0	1446	1	1	0.1
	Not vaccinated	290	2	2	0.7	344	1	1	0.3	233	0	0	0.0
51	AS04-HPV-16/18	1606	8	26	1.6	1586	9	19	1.2	-	-	-	-
	HBV	203	2	2	1.0	191	2	3	1.6	1446	10	27	1.9
	Not vaccinated	290	5	6	2.1	344	3	4	1.2	233	3	4	1.7
52	AS04-HPV-16/18	1606	4	9	0.6	1586	5	5	0.3	-	-	-	-
	HBV	203	2	2	1.0	191	2	2	1.0	1446	5	9	0.6
	Not vaccinated	290	2	2	0.7	344	3	4	1.2	233	3	3	1.3

		Arm A				Arm B				Arm C			
**HPV Type**	**Group**	**N**	**n+**	**n**	**%**	**N**	**n+**	**n**	**%**	**N**	**n+**	**n**	**%**
53	AS04-HPV-16/18	1606	8	16	1.0	1586	7	12	0.8	-	-	-	-
	HBV	203	1	1	0.5	191	4	5	2.6	1446	6	14	1.0
	Not vaccinated	290	4	4	1.4	344	2	2	0.6	233	3	3	1.3
54	AS04-HPV-16/18	1606	4	4	0.2	1586	3	4	0.3	-	-	-	-
	HBV	203	0	0	0.0	191	0	0	0.0	1446	3	4	0.3
	Not vaccinated	290	1	1	0.3	344	1	1	0.3	233	0	0	0.0
56	AS04-HPV-16/18	1606	8	19	1.2	1586	9	17	1.1	-	-	-	-
	HBV	203	1	2	1.0	191	0	0	0.0	1446	7	17	1.2
	Not vaccinated	290	1	1	0.3	344	2	2	0.6	233	3	3	1.3
58	AS04-HPV-16/18	1606	5	6	0.4	1586	3	5	0.3	-	-	-	-
	HBV	203	0	0	0.0	191	0	0	0.0	1446	4	4	0.3
	Not vaccinated	290	0	0	0.0	344	1	1	0.3	233	1	1	0.4
59	AS04-HPV-16/18	1606	2	2	0.1	1586	5	7	0.4	-	-	-	-
	HBV	203	1	1	0.5	191	1	1	0.5	1446	6	11	0.8
	Not vaccinated	290	1	1	0.3	344	1	1	0.3	233	2	2	0.9
66	AS04-HPV-16/18	1606	6	9	0.6	1586	7	13	0.8	-	-	-	-
	HBV	203	3	3	1.5	191	1	1	0.5	1446	7	9	0.6
	Not vaccinated	290	3	4	1.4	344	2	2	0.6	233	2	2	0.9
68	AS04-HPV-16/18	1606	3	5	0.3	1586	1	3	0.2	-	-	-	-
	HBV	203	1	1	0.5	191	1	1	0.5	1446	3	5	0.3
	Not vaccinated	290	0	0	0.0	344	1	1	0.3	233	2	2	0.9
70	AS04-HPV-16/18	1606	0	0	0.0	1586	1	1	0.1	-	-	-	-
	HBV	203	0	0	0.0	191	1	1	0.5	1446	0	0	0.0
	Not vaccinated	290	0	0	0.0	344	1	1	0.3	233	0	0	0.0
74	AS04-HPV-16/18	1606	4	4	0.2	1586	2	2	0.1	-	-	-	-
	HBV	203	0	0	0.0	191	0	0	0.0	1446	1	1	0.1
	Not vaccinated	290	0	0	0.0	344	1	1	0.3	233	0	0	0.0

AS04-HPV-16/18, AS04-adjuvanted HPV-16/18 vaccine; HBV, hepatitis B virus vaccine; N, number of subjects with available results; n+, number of communities with at least one event; n, number of subjects reporting an event.
